# Novel Inhibitory Effect of *N*-(2-Hydroxycyclohexyl)valiolamine on Melanin Production in a Human Skin Model

**DOI:** 10.3390/ijms150712188

**Published:** 2014-07-09

**Authors:** Bum-Ho Bin, Yung Hyup Joo, Ai-Young Lee, Song Seok Shin, Eun-Gyung Cho, Tae Ryong Lee

**Affiliations:** 1 AmorePacific Corporation R&D Center, Yongin, Gyeonggi-do 446-729, Korea; E-Mails: bbh82429@ amorepacific.com (B.-H.B.); yhjoo@amorepacific.com (Y.H.J.); ssshin@amorepacific.com (S.S.S.); 2 Department of Dermatology, Dongguk University Ilsan Hospital, 814 Siksa-dong, Ilsandong-gu, Goyang-si, Gyenoggi-do 410-773, Korea; E-Mail: leeay@duih.org

**Keywords:** valiolamine derivative, melanin, melanogenesis, tyrosinase

## Abstract

Hyper-pigmentation causes skin darkness and medical disorders, such as post-inflammatory melanoderma and melasma. Therefore, the development of anti-melanogenic agents is important for treating these conditions and for cosmetic production. In our previous paper, we demonstrated that the anti-diabetic drug voglibose, a valiolamine derivative, is a potent anti-melanogenic agent. In addition, we proposed an alternative screening strategy to identify valiolamine derivatives with high skin permeability that act as anti-melanogenic agents when applied topically. In this study, we synthesized several valiolamine derivatives with enhanced lipophilicity and examined their inhibitory effects in a human skin model. *N*-(2-hydroxycyclohexyl)valiolamine (HV) possesses a stronger inhibitory effect on melanin production than voglibose in a human skin model, suggesting that HV is a more potent anti-melanogenic agent for the skin.

## 1. Introduction

Melanin, a natural pigment, is a complex polymer synthesized by living organisms [1]. Melanin biosynthesis is initiated from the hydroxylation of an aromatic amino acid l-tyrosine to l-dihydroxyphenylalanine (l-DOPA) [1,2]. l-DOPA is then oxidized to dopaquinone by tyrosinase, which is the rate-limiting step in melanin biosynthesis, followed by dopachrome tautomerization and serial oxidation steps [1,2]. 

Melanin plays a critical role in protecting cells from cytotoxic UV irradiation and is largely responsible for human skin color [3,4,5]. However, the over-production of melanin causes several clinical problems, including post-inflammatory melanoderma and melasma and skin darkness [6,7,8]. Therefore, researchers have studied anti-melanogenic agents to treat hyper-pigmentation conditions. In a previous paper, we reported that the *N*-substituted valiolamine derivative voglibose demonstrated a strong inhibitory effect on melanin production by inhibiting proper *N*-glycan processing of tyrosinase, resulting in a dramatic reduction of tyrosinase protein levels by altering its stability [9]. Originally used as an oral anti-hyperglycemic agent that acted on intestinal surface enzymes, voglibose was systemically synthesized to have low lipophilicity [10]. In contrast to oral anti-hyperglycemic drugs, anti-melanogenic agents must be cell-permeable to operate inside the cell. Therefore, we proposed an alternative strategy to discover anti-melanogenic agents that are more effective than voglibose by screening valiolamine derivatives with increased lipohilicity [9]. As part of our continuing efforts to develop an effective anti-melanogenic agent, we synthesized valiolamine derivatives with higher lipophilicity than voglibose and selected a derivative that did not affect cell growth. The inhibitory effect of this derivative on melanin production was evaluated.

## 2. Results and Discussion

### 2.1. N-(Trans-2-hydroxycyclohexyl)valiolamine Treatment Reduces Tyrosinase Protein Levels

**Figure 1:**
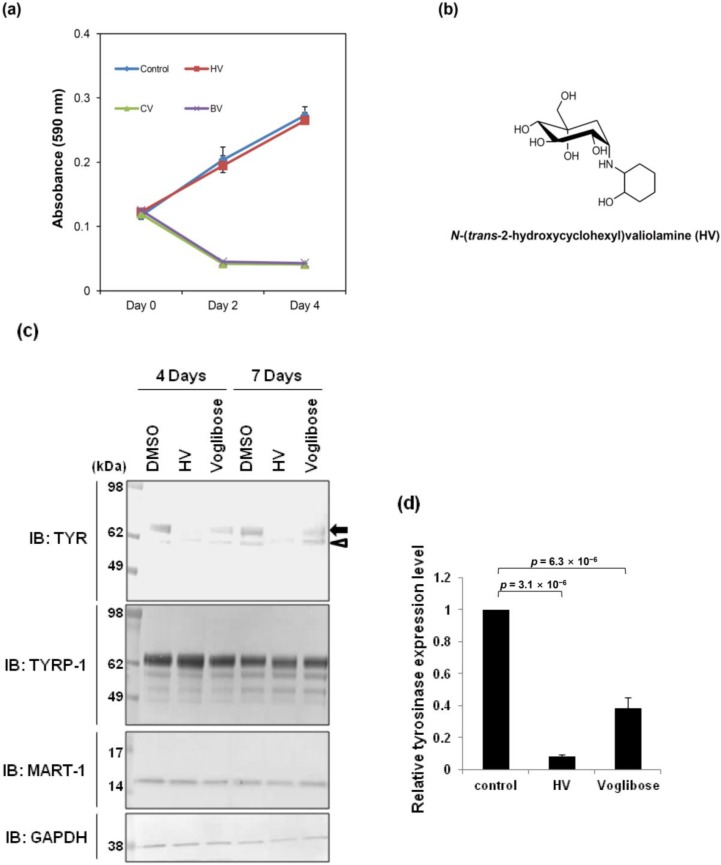
HV (*N*-(2-hydroxycyclohexyl)valiolamine) reduces the expression of tyrosinase protein. (**a**) Cell growth curves after treatment with each valiolamine derivative. Cells were treated with 2 mM *N*-cyclohexylvaliolamine (CV), *N*-(4-bromobenzyl)valiolamine (BV) or *N*-(trans-2-hydroxycyclohexyl)valiolamine (HV), followed by a cell growth assay. The data are representative of three independent experiments; (**b**) HV structure; (**c**) Melanogenesis-related protein expressions in normal human melanocytes before and after treatment with 2 mM HV or voglibose. Cells were cultured with 2 mM HV or voglibose for the indicated periods, and the cell extract was analyzed by immunoblotting using each indicated antibody. Fully *N*-glycosylated mature (arrow) and immature (arrowhead) tyrosinase bands are indicated; and (**d**) The relative tyrosinase levels were analyzed using Image J software [12] with Western blot image after seven days of treatment. The graph is representative of two independent experiments. Data are shown as mean ± SEM.

In our previous paper, we demonstrated that voglibose inhibits melanogenesis in reconstructed human skin and proposed a strategy for screening valiolamine derivatives to discover effective anti-melanogenic agents with increased cell permeability [9]. The cell permeability of a compound can be estimated by its cLogP value, which represents the logarithm of a compound’s partition coefficient between n-octanol and water [log (c_octanol_/c_water_)]; the cLogP value is a well-established index for a compound’s lipophilicity [11]. High cLogP values are indicative of high cell permeability. Following the suggested process [10], three valiolamine derivatives with increased cLogP values compared with voglibose [*N*-(trans-2-hydroxycyclohexyl)valiolamine (HV), −0.14; *N*-cyclohexylvaliolamine (CV), 0.95; *N*-(4-bromobenzyl)valiolamine (BV), 1.50; voglibose, −2.34] were synthesized. These derivatives also showed comparable inhibitory effect on α-glucosidases to voglibose [10]. We initially tested cell toxicity by observing the growth of human melanocytes in the presence of 2 mM of each compound (Figure 1a). Only HV did not affect cell growth (Figure 1a,b, HV); therefore, we used this compound for further study. Next, we investigated the effects of HV on the expression of tyrosinase because the valiolamine derivative voglibose reduced melanin production by down-regulating tyrosinase protein levels [9]. Melanocytes were seeded and cultured with 2 mM HV or voglibose for four and seven days. In dimethyl sulfoxide (DMSO)-treated cells, tyrosinase was detected as two bands, fully *N*-glycosylated mature (arrow) and immature (arrowhead) forms (Figure 1c, DMSO). As shown in our previous report, voglibose treatment reduced fully *N*-glycosylated mature tyrosinase protein levels (Figure 1c, Voglibose) [9]. Similarly, HV treatment also reduced fully modified mature tyrosinase protein levels, but the effect was more severe than with voglibose (Figure 1c,d, HV), suggesting that HV is a more potent anti-melanogenic agent. We observed that tyrosinase-related protein (TYRP-1) and melanoma antigen recognized by T-cells 1 (MART-1) were not altered after HV treatment, similar to voglibose treatment (Figure 1c) [9]. These results indicate that the stability of tyrosinase is more sensitive to its glycosylation state than to that of other melanogenesis-related glycoproteins, as discussed in our previous report [9].

### 2.2. HV (N-(2-Hydroxycyclohexyl)valiolamine) Treatment Reduces Melanin Production in a Reconstructed Human Skin Model

**Figure 2:**
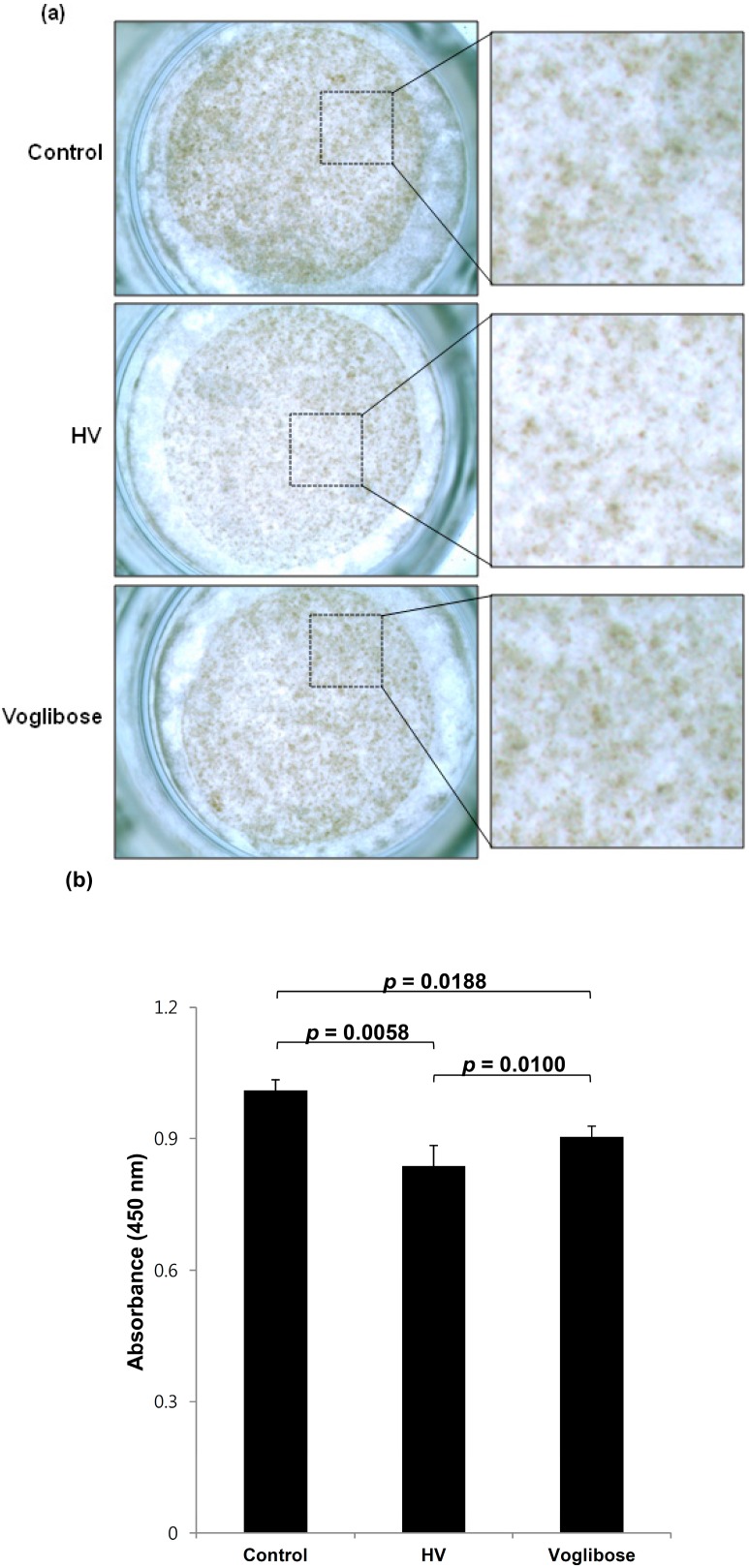
The inhibitory effect of HV on melanin production in a reconstructed human skin model. (**a**) The reconstructed human skin samples were irradiated with 20 mJ/cm^2^ ultraviolet B (UVB) every other day for a total of four exposures and simultaneously treated with 2 mM HV or voglibose for 10 days. Each representative image was obtained after treatment. The insets show the magnified images; (**b**) The melanin content of the lysates was measured at 450 nm after dissolving the reconstructed human skin samples in 1 N NaOH. Data are shown as mean ± SEM (*n* = 3).

To verify the effect of HV on melanin production in human skin, we irradiated reconstructed human skin with 20 mJ/cm^2^ ultraviolet B (UVB) every other day for a total of four UVB exposures and simultaneously treated the skin with 2 mM voglibose or HV for 10 days as described previously [9]. The visual and spectrophotometric evaluations revealed a reduction of melanin production in voglibose-treated reconstructed human skin [9]. HV exhibited a stronger inhibitory activity on melanin production than voglibose (Figure 2a). Voglibose- and HV-treated cells showed 90.3% ± 2.5% and 83.6% ± 4.8% total melanin content compared with control (100%), respectively (Figure 2b). These results indicate that HV is a potent anti-melanogenic agent and that valiolamine derivatives are good candidates for developing anti-melanogenic agents. 

Since the mid-1970s, pseudo-oligosaccharides purified from bacteria have received attention as potential drugs for treating type 2 diabetes mellitus [13]. *N*-substituted valiolamine derivatives, including voglibose, were systemically synthesized and tested as oral anti-hyperglycemic agents. This class of drug was developed with low cell membrane permeability because these drugs act on intestinal surface enzymes [10]. In contrast to oral anti-hyperglycemic drugs, anti-melanogenic agents must be cell-permeable to act on intracellular α-glucosidases. Based on the fact that HV has a higher cLogP value than voglibose, indicative of higher cell permeability, we performed dose response curves comparing HV to voglibose in melanocyte monolayer cultures. However, we could not observe any significant differences in a dose-dependency between them. We reasoned that in monolayer cultures, if both compounds are strong enough to show the effect, it may be difficult to discriminate the efficacy between them. In spite of the limitation in monolayer culture, we did observe that the protein level of tyrosinase was more severely reduced in melanocytes treated with HA than the same amount of voglibose (Figure 1c). Considering of the higher cLogP value and the stronger effects on skin equivalent and tyrosinase level, we think that the higher permeability is one of important reasons for HA to show the superior efficacy than voglibose.

Here, we report that HV has increased cLogP and enhanced inhibition of melanin production compared with voglibose and suggest HV as a potential treatment for hyperpigmentary skin conditions. Whether other numerous valiolamine derivatives synthesized during the course of voglibose development serve as additional anti-melanogenic agents remains to be investigated.

## 3. Experimental Section

### 3.1. Cell Culture

Moderately pigmented normal human melanocytes (Cascade Biologics, Portland, OR, USA) were maintained in M-254 medium (Cascade Biologics) supplemented with human melanocyte growth supplement (Cascade Biologics).

### 3.2. Chemical Compounds Synthesis

Valiolamine derivatives were prepared as previously reported [10]. cLogP values for compounds were calculated by Chem Draw Ultra 7.0.1. (Cambridgesoft, Cambridge, MA, USA).

### 3.3. Melanin Assay

Neoderm-ME (Tego Science, Seoul, Korea) containing normal human melanocytes and keratinocytes was purchased and maintained as previously described [9]. Briefly, Neoderm-ME was removed from the 12-well plates containing agarose and placed in 6-well plates containing 3 mL of the medium provided by the manufacturer. Neoderm-ME was irradiated with 20 mJ/cm^2^ UVB every other day for a total of four UVB exposures, and simultaneously, the skin was treated with 2 mM of the compounds for 10 days. After Neoderm-ME was dissolved in 1 N NaOH and sonicated, the debris was separated by centrifugation at 16,000× *g* for 1 min. The absorbance at 450 nm was measured from supernatants to determine melanin content.

### 3.4. Cell Growth Assay

The moderately pigmented normal human melanocytes were seeded at a density of 2 × 10^4^ per well in 6-well plates. On each day of assay, cell fixation was performed with 4% paraformaldehyde in phosphate buffered saline (PBS) for 15 min. Then, the cells were washed with PBS and stained with 500 µL of 0.1% crystal violet for 10 min. The stained cells were dried for 5 min and lysed with 1 mL of 10% acetic acid. Absorbance at 590 nm was measured to determine the rate of cell growth.

### 3.5. Western Blot Analysis

Cells were collected in 1% NP-40 containing 0.05 M Tris-HCl, pH 7.5, 0.15 M NaCl, and 0.01 M MgCl_2_. After centrifugation at 16,000 rpm for 20 min, the supernatants were separated. For sodium dodecyl sulfate polyacrylamide gel electrophoresis (SDS-PAGE), these fractions were boiled for 5 min in SDS-PAGE sample buffer containing 0.125 M Tris-HCl, pH 6.8, 20% glycerol, 4% SDS, 10% 2-mercaptoethanol, and 0.004% bromophenol blue (BPB). Then, 20 µg of protein was loaded onto a 4%–12% gradient gel. For immunoblotting, the gel was electroblotted to a polyvinylidene fluoride (PVDF) membrane, which was blocked in 5% skim milk. Anti-GAPDH antibody (Santa Cruz Biotechnology, Santa Cruz, CA, USA), anti-TYRP-1 antibody (Santa Cruz Biotechnology), anti-MART-1 antibody (Thermo Fisher Scientific, San Jose, CA, USA), and anti-tyrosinase antibody (Upstate Biotechnology, Lake Placid, NY, USA) were used for protein detection.

### 3.6. Statistical Analysis

A two-tailed Student *t* test was used to analyze differences between the two groups.

## 4. Conclusions

*N*-(Trans-2-hydroxycyclohexyl)valiolamine (HV) is a potentially effective anti-melanogenic agent.
